# Review on bovine respiratory syncytial virus and bovine parainfluenza – usual suspects in bovine respiratory disease – a narrative review

**DOI:** 10.1186/s12917-021-02935-5

**Published:** 2021-07-31

**Authors:** Birgit Makoschey, Anna Catharina Berge

**Affiliations:** 1 Intervet International BV/MSD-Animal Health, Wim de Körverstraat, 5831AN Boxmeer, The Netherlands; 2 Department of Reproduction, Obstetrics and Herd Health, Faculty of Veterinary Medicine, Ghent University, 9820 Merelbeke, Belgium

**Keywords:** Bovine respiratory syncytial virus, BRSV, Bovine Parainfluenza, BPIV3, Bovine respiratory disease, BRD, Vaccines, Review

## Abstract

Bovine Respiratory Syncytial virus (BRSV) and Bovine Parainfluenza 3 virus (BPIV3) are closely related viruses involved in and both important pathogens within bovine respiratory disease (BRD), a major cause of morbidity with economic losses in cattle populations around the world. The two viruses share characteristics such as morphology and replication strategy with each other and with their counterparts in humans, HRSV and HPIV3. Therefore, BRSV and BPIV3 infections in cattle are considered useful animal models for HRSV and HPIV3 infections in humans.

The interaction between the viruses and the different branches of the host’s immune system is rather complex. Neutralizing antibodies seem to be a correlate of protection against severe disease, and cell-mediated immunity is thought to be essential for virus clearance following acute infection. On the other hand, the host’s immune response considerably contributes to the tissue damage in the upper respiratory tract.

BRSV and BPIV3 also have similar pathobiological and epidemiological features. Therefore, combination vaccines against both viruses are very common and a variety of traditional live attenuated and inactivated BRSV and BPIV3 vaccines are commercially available.

## Background

Bovine Respiratory Disease (BRD) affects young calves and young stock in all parts of the world and accounts for considerable economical losses. Outbreaks are typically related to environmental stress factors (transport, crowding, unfavorable climate conditions) as the disease results from the interactions between microorganisms in the respiratory tract and the animal’s resistance, which is affected by such non-biological factors. The clinical picture is characterized by respiratory symptoms and the severity can range from mild to severe with sometimes even fatal outcomes. Co-infection with several pathogens is the rule rather than the exception. Bovine Respiratory Syncytial Virus (BRSV) and Bovine Parainfluenza 3 Virus (BPIV3) are two closely related viruses that are often involved in BRD outbreaks. Having a lot of similarities in their morphology and replication strategy, also the (patho-)biology of the two viruses has a lot of common features. Intense research has led to the development of vaccines against the two viruses, often as bi-valent vaccines or in combination with other respiratory pathogens. Herein, the similarities and differences between BRSV and BPIV3 are presented to eventually provide a better understanding of their role and importance in the BRD complex.

## Main text

### The viruses: two close relatives

BRSV and BPIV3, along with their human counterparts HRSV and HPIV3, belong to the order Mononegavirales. Viruses belonging to this order are characterized as enveloped viruses with non-segmented, single-stranded, negative-sense RNA genomes. Formerly, these viruses were classified as Paramyxoviridae, but in 2016 the family Pneumoviridae was created and nowadays BRSV and HRSV belong to the genus Orthopneumovirus within this family. BRSV is therefore also referred to as Bovine orthopneumovirus [1, 2]. The official name of BPIV3 is Bovine respirovirus 3 as the virus is classified into the Respirovirus genus within the Paramyxoviridae family. Other members of this genus are the antigenically and genetically related human parainfluenza virus types 1 and 3 (HPIV1 and HPIV3, respectively) [3].

**Fig. 1:**
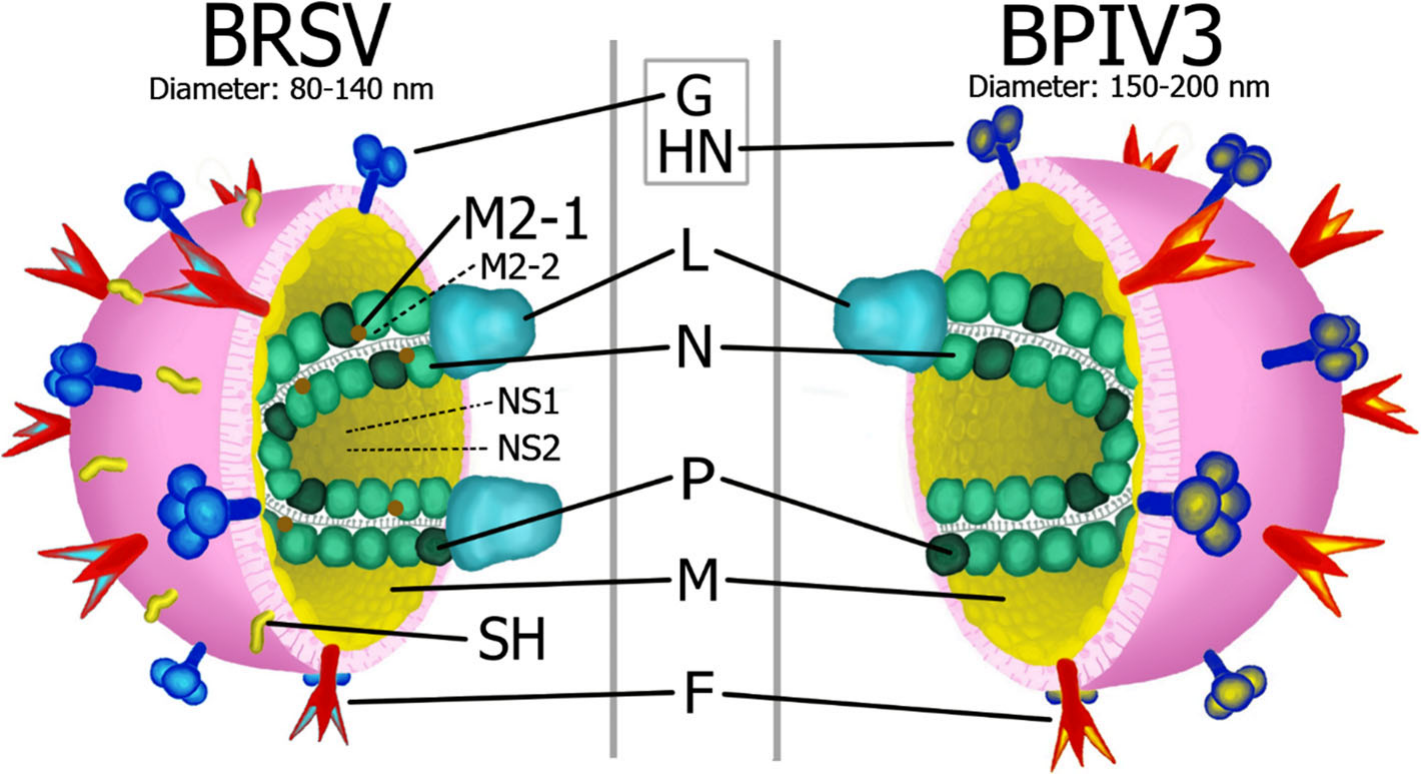
Morphology of BRSV and BPIV3. G: G protein; HN: Haemagglutinase – Neuraminidase; L: Polymerase protein; M: Matrix protein; N: Nucleoprotein; P: Phosphoprotein; F: Fusion protein

BRSV and BPIV3 have a spherical to pleomorphic shape at a size of 150–200 nm (Fig. 1). The virions consist of a nucleocapsid surrounded by a lipid envelope which is directly derived from the host cell membrane by budding. Viral transmembrane glycoproteins are located on the surface of the envelope. The major attachment protein G of BRSV is synthesized as two forms, a membrane-anchored and a secreted form, and around 80% of the G protein is produced as the secreted form 24 h after infection [4]. In addition to its role in the attachment to host cells, the G-protein may have other roles such as interacting with the immune system. It has been proposed that the secreted form might act as a decoy by binding to neutralizing antibodies [5].

Recombinant BRSV lacking the G protein is still competent to replicate in cell culture as well as in young calves and to induce a protective immune response [6, 7].

**Fig. 2:**
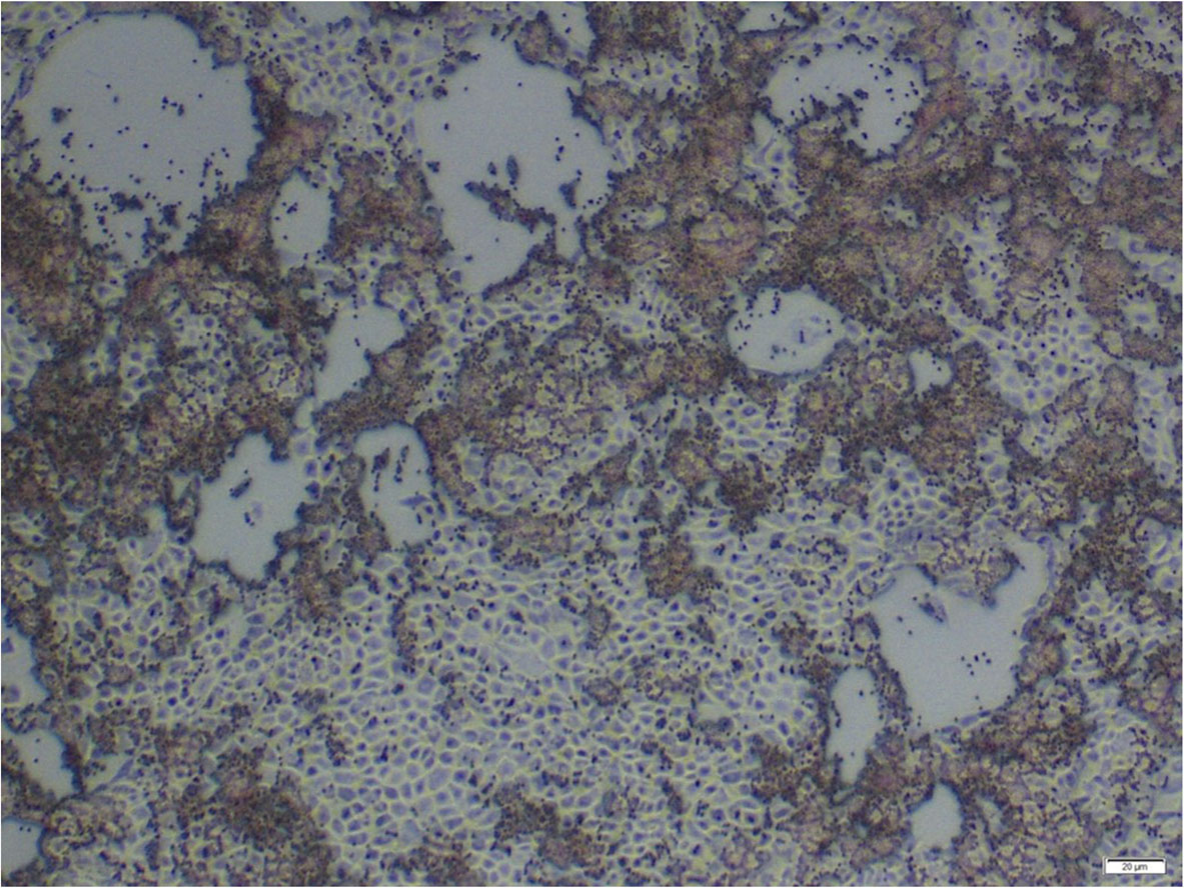
Cytopathic effect caused by BPIV3 virus and haemadsorption of erythrocytes onto the surface of the infected cell layer

The counterpart of the BRSV G protein in BPIV3 and other Paramyxoviruses has haemagglutinating properties (Fig. 2), it is called the haemagglutinin-neuraminidase (HN) protein. The BRSV G and BPIV3 HN proteins bind to sialic acid residues present on cell surfaces throughout the respiratory tract. Chimeric recombinant BRSV in which the G protein was replaced by the HN of BPIV3 are replication competent in vitro, although the two glycoproteins differ considerably in sequence and structure [8].

The BRSV G and BPIV3 HN proteins, together with the fusion protein (F), which both viruses have in common, mediate attachment and entry of the virions into the cells and delivery of the nucleocapsid into the cytoplasm of the host cell.

BRSV has a third glycoprotein, the small hydrophobic protein (SH). This protein has ion-channel functions [9] and may play a role in virus mediated cell fusion by interacting with the F protein [10]. SH has been shown to be non-essential for growth in vitro and in vivo, but mutants lacking the SH protein were attenuated [11].

The helical nucleocapsid consist of the nucleoprotein (N), the phosphoprotein (P), the viral RNA-dependent polymerase protein (L), and the genomic RNA of around 15,000 nucleotides in length.

The non-glycosylated matrix protein M is located on the inner envelope of the capsid and is the most abundant protein in infected cells. The M proteins are involved in assembly, budding and release of progeny viruses [12]. Different from other Paramyxoviridae, BRSV and other pneumoviruses have two additional matrix proteins, M2–1 and M2–2 which play a role in virus replication [13, 14].

Another major difference between the pneumoviruses (such as BRSV and HRSV) and the other Paramyxoviridae is the presence of two nonstructural (NS) proteins, NS1 and NS2. There is evidence that the NS proteins are inhibitors of viral RNA transcription and replication and they cooperatively antagonize the antiviral alpha/beta interferon-induced response of the host cell [15, 16]. These proteins are not essential for virus replication in vitro, although recombinant BRSV lacking NS1 or NS2 was severely attenuated in IFN-competent cells and in young calves [17].

Transcription of the negative-sense genomic template occurs in the cytoplasm of host cells. This generates sub-genomic positive-sense mRNAs. These are then copied into a full-length negative-sense antigenomic RNA which is encapsidated. The resulting ribonucleoprotein complex is transported to the cellular surface membrane, where budding occurs [18].

A characteristic that BRSV and BPIV3 share with other single-stranded RNA viruses is the high mutation rate, which confers a high adaptability of the virus. Analysis of the genetic evolution of a BRSV isolate during in vitro passage and subsequent inoculation in calves suggest that virus populations may evolve as complex and dynamic mutant swarms, although they appear to be genetically stable [19]. This likely depends on the type of cells and number of passages and may explain, why a loss of virulence following in vitro passage was observed in some infection studies [20], but not in others [21].

Another consequence of the high mutation rate is the antigenic variation. The BRSV has been classified in four antigenic groups (A, B, AB and an intermediate group) with six genetic groups. A continuous evolution occurs mainly in the antigenically important G-protein, especially in geographical regions where vaccines are widely used [22]. Studies with a new intranasal live BRSV – BPIV3 vaccine have demonstrated cross protection against BRSV isolates from a different geographic origin [23].

The antigenic variation in BPIV3 viruses appear to be less important than in BRSV. Phylogenetic reconstructions based on the nucleotide sequences for the M-protein and the entire genome, demonstrated two distinct BPIV-3 genotypes (BPIV-3a and BPIV-3b) and more recently, a third genotype [24, 25] has first been described in China and it seems to geo-expand sinceBPIV-3c has recently been reported in Serbia [26] and Turkey [27].

### Epidemiological features – infection dynamics within and between herds

Like other respiratory viruses, BRSV and BPIV3 virus are horizontally transmitted. Airborne transmission of BRSV has been reproduced under experimental conditions [28]. This route, in combination with regular sub-clinical re-infection, is considered the main mechanism for spreading of BRSV and BPIV3 within a herd [29, 30]. Persistence of BRSV in cattle has been proposed [31, 32], but attempts to reactivate putative persistent BRSV were not successful [33]. Moreover, a Danish study showed up to 11% of genetic diversity between BRSV from various outbreaks in a herd, which is suggestive for a re-introduction of a virus into a herd rather than a latent re-circulating virus [34].

Also, in the case of BPIV3 it has been suggested that subclinical infections contribute to the maintenance of the infection in cattle populations [30].

Inter-herd transmission of BRSV is frequent and the virus has been detected in outbreaks in the summer months too, indicating that virus circulation occurs throughout the year [35]. The clustering of BRSV sequences according to geographical origin [22] might suggest a role for airborne transmission or mechanical transmission by visitors such as veterinarians.

### Pathogenesis - what happens when the viruses get into the animal?

**Fig. 3:**
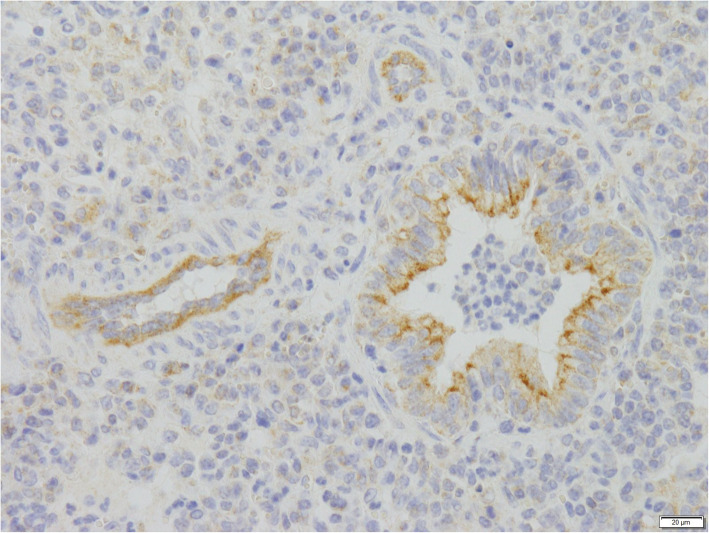
Formation of syncytia and immunostaining of BRSV in mucosal cells in the bronchioli of a calf infected with BRSV

BRSV and BPIV3 are mainly spread by air droplet transmission and they enter the body via the respiratory tract. Once inhaled, the viruses penetrate or possibly degrade the mucous, which is the first line of defense of the innate immune system and thereafter invade epithelial cells of the upper respiratory tract by binding to sialic acid residues on the cell membranes. BRSV and BPIV3 replicate predominantly in the respiratory tract [36, 37]. Infected animals excrete virus with nasal discharge during several days. In general, BRSV reaches lower titers than BPIV3 virus, both in tissue culture and after infection of animals [38]. Both viruses have been shown to infect tracheal cells, ciliated and non-ciliated bronchiolar cells (see Fig. 3), as well as pneumocytes [36, 39]. In contrast to BRSV, BPIV3 also invades and multiplies well in pulmonary alveolar macrophages (PAM). The replication of BPIV3 in the PAM has been linked to depression of phagocytosis and immunosuppressive prostaglandins [40]. Lymphocyte proliferation seems to be suppressed by bovine alveolar macrophages infected with the virus [41].

In continuous cell lines, both viruses grow with cytopathic effects characterized by the formation of syncytia. In an BRSV infected animal, pro-inflammatory genes are upregulated [42] and extensive mast-cell degeneration was observed in peracute cases of BRSV-related disease [43]. These findings suggest that the host’s immune response causes further tissue damage and contributes to the lung pathology [10, 44]. Antonis and colleagues [45] investigated age-dependent differences in the pathogenesis of BRSV infection in antibody negative calves at the age of 1 day or 6 weeks. Neonatal calves had more extensive virus replication and lung consolidation, but lower pro-inflammatory responses, specific humoral immune responses, lung neutrophilic infiltration and clinical signs in comparison with 6-week-old calves. The capacity to produce pro-inflammatory cytokines appeared to increase with age and could therefore explain the observed age-dependent differences in the pathogenesis of BRSV.

Also, for BPIV3 virus, the immune response seems to be involved in the pathogenesis as supported by the finding that increased levels of histamine were released from mast cells from the lungs of BPIV3-infected calves [46]. Moreover, transcription of cytokines related to fever and other signs of inflammation like TNFα, IL1β, and IL6 were found to be upregulated after infection with BPIV3 [47].

The resulting tissue damage in combination with the host’s pro-inflammatory response favour secondary bacterial infections [48] leading to aggravation of the disease [30, 49–51].

### Clinical symptoms and lesions

The severity of BRD symptoms can range from sub-clinical to fatal outcomes [52–54] depending on different factors such as the age, and immunological status, the presence of specific antibodies and immunosuppression. Under experimental conditions, the severity of disease might be related to the route and dose of infection as well as the virulence of a particular strain or the degree of attenuation following culture in vitro [20, 30], while under field conditions co-infection with other pathogens have an influence on the severity.

In the field, it is difficult to attribute the symptoms and lesions of a BRD case to a single pathogen, but information is available from experimental infection studies with either of the two viruses.

The common clinical symptoms associated with BRSV and BPIV3 are similar. The peak in clinical signs for BRSV is usually reached 4 to 6 days after infection [55]. Naïve calves usually develop a fever starting about 2 days after exposure, with body temperatures reaching up to 40 °C. Fever is often associated with depression, lack of appetite or anorexia, and an increased respiratory rate. The airways can become obstructed through the overproduction of mucous [56] that can lead to coughing and mucopurulent nasal discharge. Upon auscultation of the lungs, wheezing can be heard. A peracute severe form of disease related to infection with BRSV has been observed in beef cattle [43], but most animals recover within 10 days, unless other respiratory pathogens are involved [30, 57]. Clinical disease symptoms due to BPIV3 infections have been described as less severe compared to BRSV [58].

**Fig. 4:**
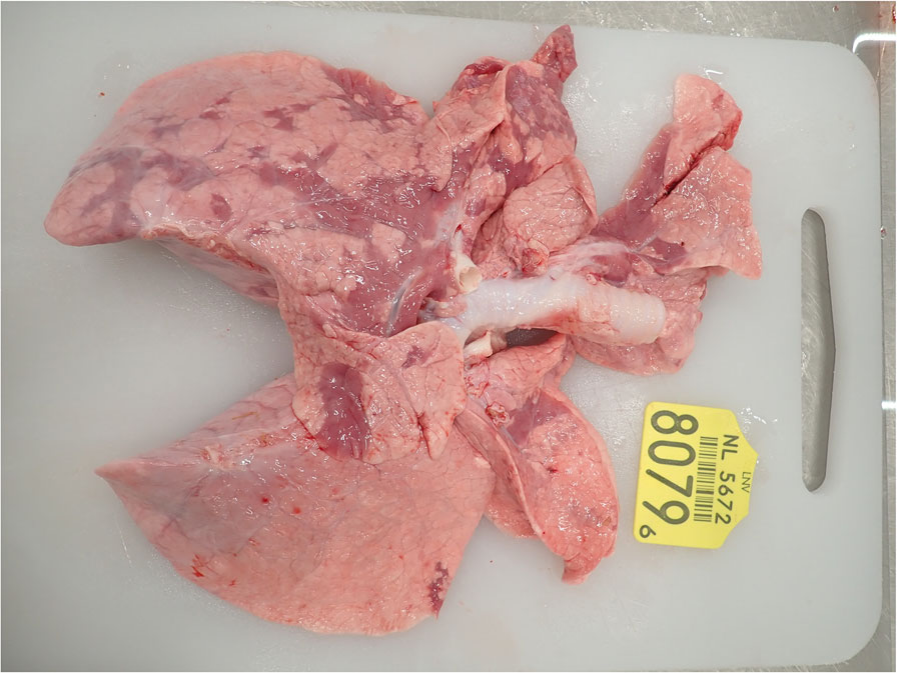
Interstitial pneumonia in a lung of a calf two weeks after infection with BRSV

The results obtained at post-mortem investigation are similar for both viruses. The most common macroscopic lesions described are multilobular consolidation (Fig. 4), mainly in the cranial lung lobes. Interlobular emphysema may be seen after infection with BRSV but has not been described after infection with BPIV3.

Histological lesions associated with both viruses include bronchiolitis with peribronchial mononuclear infiltrates, epithelial necrosis and syncytia formation (Fig. 3) [30, 59, 60].

### Interactions between the viruses and the immune system

#### Modulation of the innate immune response

BRSV infection in calves is considered an ideal model to study the pathogenesis of HRSV [61]. In this context, the complex interactions between the virus and the innate immune system have been extensively studied [62, 63]. Considerably less information is available for BPIV3.

BRSV activates the innate immune response resulting in the induction of a variety of pro-inflammatory cytokines and chemokines which contributes to the pathology [10, 44, 45]. Moreover, it has been shown that BRSV can modulate the innate and adaptive immune response to mitigate stimulation of a CD8+ T cytotoxic cell response and instead promote a Th2 response [63].

The NS1 and NS2 proteins of human and bovine RSV suppress the induction of type 1 Interferon (IFN), one of the major anti-viral defence mechanisms of the innate immune system. In addition, the NS proteins provide resistance of the virus to the anti-viral effects of type I IFNs [15]. The G protein can also modulate components of the innate and adaptive immune response, leading to a reduction in the BRSV specific immune response [10, 64]. These immunomodulating properties might explain why deletion of the genes coding for either of the two proteins leads to attenuation in calves [7, 11]. The failure of natural infection to prevent re-infection [65] might be related to the capacity of BRSV to suppress the host’s immune response.

**Fig. 5:**
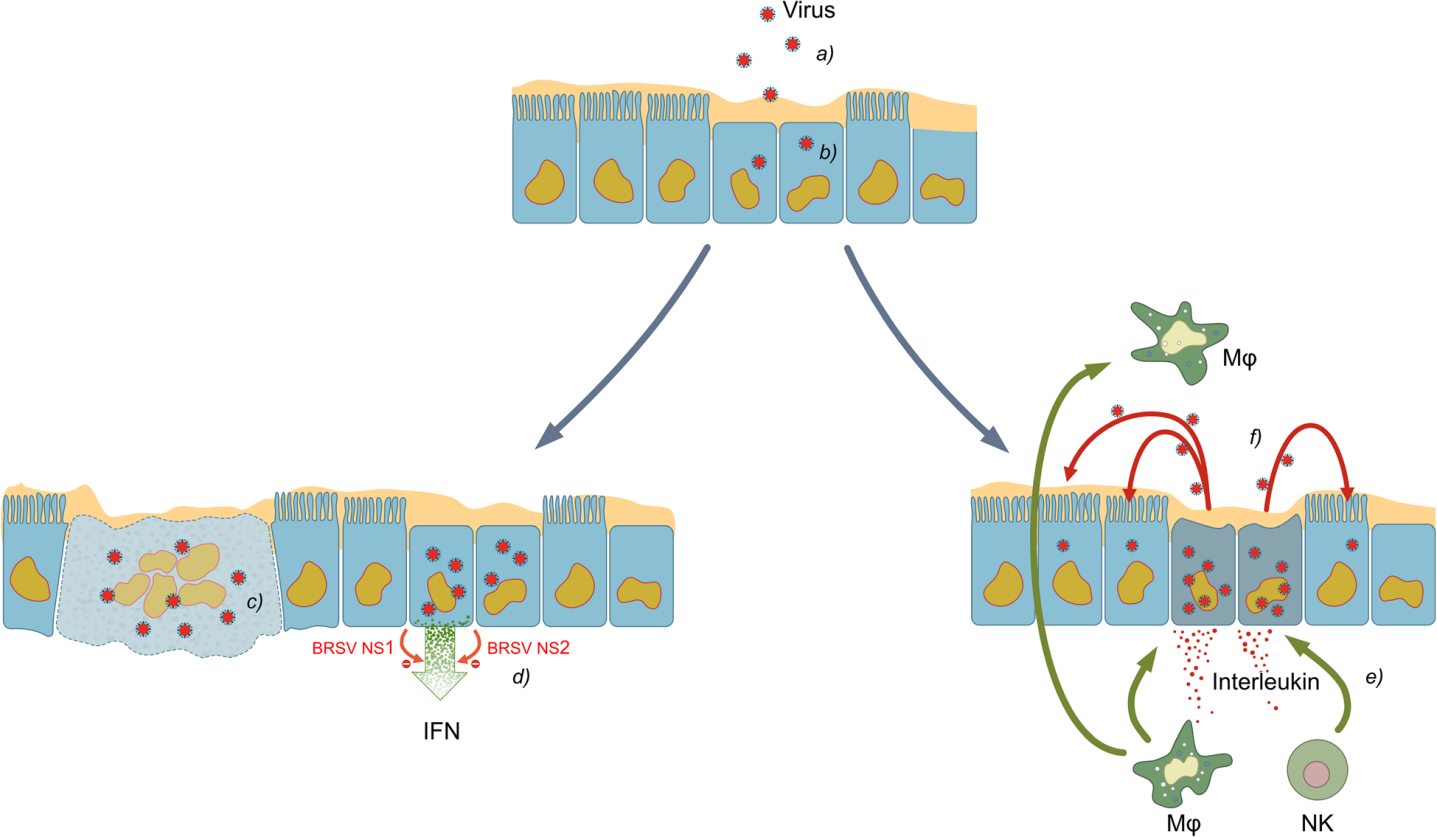
Virus infection and interaction with the innate immune system. **a** Virus passes the mucous layer, binds to sialic acids on the surface of the cell membrane. **b** Virus and enters the epithelial cell. **c** The virus replication in the infected cells leads to damage of the cells and to the formation of syncytia (cytopathic effect). **d** The virus infection triggers an antiviral innate immune response (production of interferon). The NS1 and NS2 protein of BRSV reduce the interferon response. **e** Macrophages and natural killer cells are attracted and destroy virus infected cells. **f** The immune response leads to further tissue damage. BRSV and PI3 both enhance this process

The complex interaction between virus and immune system is depicted in Fig. 5.

#### Role of the adaptive immune response

**Fig. 6:**
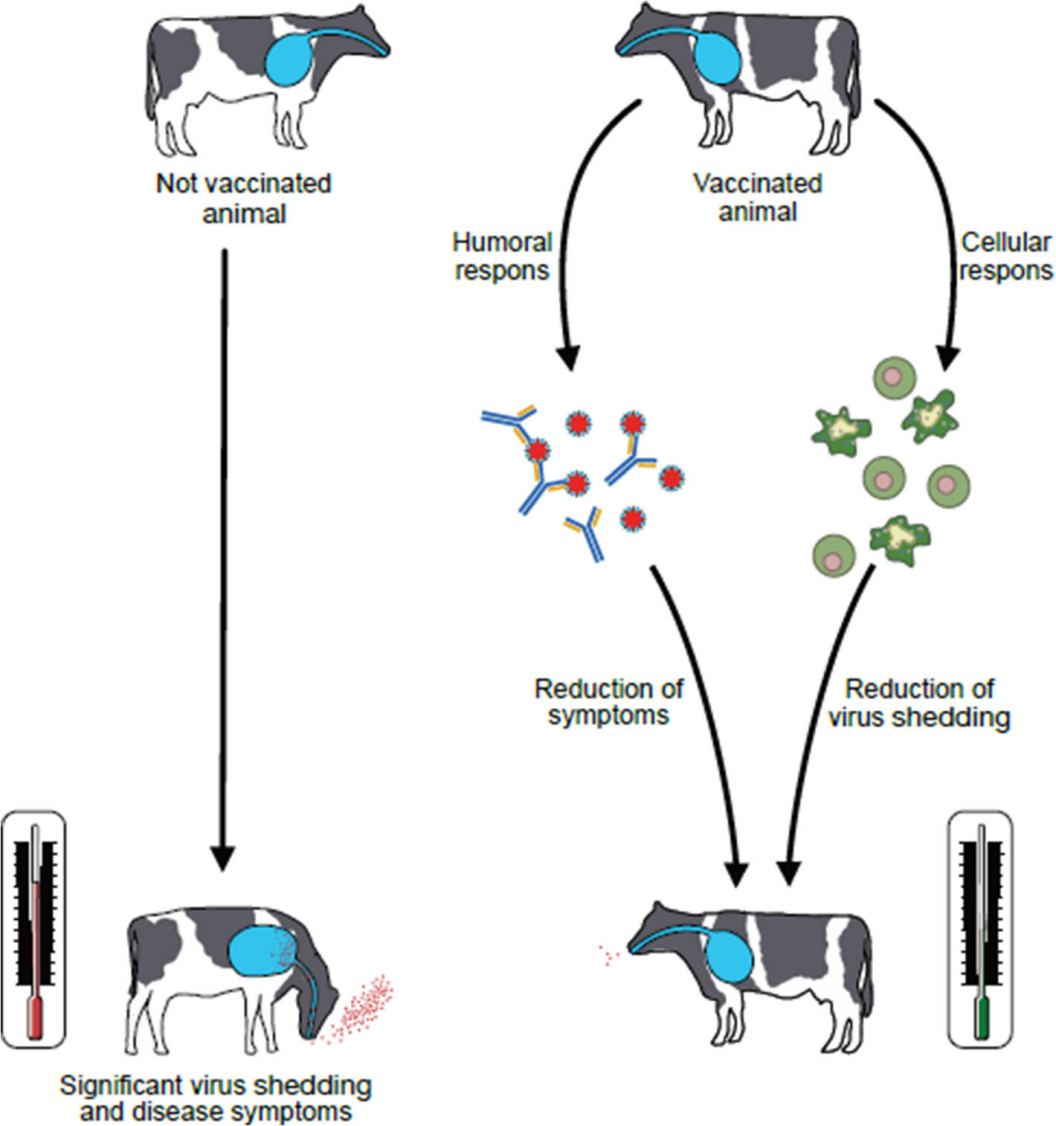
Role of humoral and cellular immune response in controling virus infection

The protective immune response against BRSV and BPIV3 involves both humoral (antibody) and cell-mediated immunity with different roles for the two branches. While neutralizing antibodies seem to be a correlate of protection against severe disease, cell-mediated immunity is considered to be essential for virus clearance following acute infection [44] (Fig. 6).

The viral proteins that have been most associated with protective antibodies are the major surface glycoproteins G and F of BRSV or HN and F of BPIV3, respectively. Infected cattle develop antibodies directed against these glycoproteins as well as some of the minor viral proteins [30, 63].

As BRSV G protein and the BPIV3 HN protein are the major attachment proteins, neutralizing antibodies directed against them prevent attachment to the cells. The inhibition of haemagglutination by HN specific antibodies contributed to the initial discovery of BPIV3 [66].

Results with HRSV in mice, suggest that G-specific antibodies might be neutralizing the virus, and might be involved in antibody-mediated cellular immune functions [67]. On the other hand, the soluble form of the HRSV G protein has been shown to antagonize antibody-mediated inhibition of virus replication [68].

Anti-F antibodies of BRSV and BPIV3 have been shown to prevent cell penetration and cell fusion [69, 70], but the F protein of BRSV also have epitopes that induce non-neutralizing antibodies, which may enhance complement activation which can be involved in the pathogenesis as well as in recovery from BRSV [71].

Both, under field and experimental conditions it has been shown that presence of neutralizing antibodies (either maternally derived or due to previous infection) does not fully prevent disease but reduces the severity of the disease, both for BRSV [72, 73] and BPIV3 [30, 74]. In an epidemiological study with antibody positive calves, neutralizing antibody levels were inversely related to the severity of disease after infection with BRSV [75]. Interestingly, intratracheal application of bovine monoclonal antibodies directed against the BRSV F protein 24 h prior to challenge infection protected the lungs of gnotobiotic calves from virus infection [70].

A humoral immune response consisting of local mucosal IgA, and systemic IgM and IgG can usually be detected within approximately 1 week after infection with BRSV or BPIV3 [75, 76]. The mucosal antibodies decline to low levels after 6 to 8 weeks, whereas the serum antibodies persist for 3 to 5 months.

A strong secondary immune response against BRSV with mucosal and systemic IgA and mucosal IgM can be seen already 6 days after infection [77].

Re-exposure with BRSV or BPIV3 results in a strong serum and mucosal antibody response. It was noted that high concentrations of mucosal antibodies protect against disease, whereas the serum antibodies reduce the severity of disease once it has occurred [30].

Colostral antibodies provide partial protection against clinical disease. On the other hand, they can hamper the induction of an active humoral immune response after infection or vaccination, but they do not suppress priming of the humoral and cellular immune system as indicated by a rapid response systemic and mucosal IgA response after secondary infection [75].

In several studies, clear antibody responses were measured after vaccination [38, 78], yet no information is available, whether these responses was directly correlated with protection. Moreover, the immune response and level of protection may differ between vaccines depending on the type of vaccine and the route. Results from efficacy studies with live BRSV and BPIV3 vaccines suggest that animals with a low or even undetectable antibody response can be protected [38, 79–81], Makoschey et al., unpublished observation) and protection seems rather be correlated to the ability to mount a rapid secondary mucosal IgA response [82] or to the cell mediated immune response [44]. Cell mediated responses can be induced by modified-live, conventional vaccines as well as some inactivated BRSV vaccines [83–85].

The mechanisms for initiation of cell mediated immunity against BRSV have been studied quite extensively and comprehensive reviews are available [44, 62]. A few viral proteins have been identified as T cell targets: Epitopes for CD4+ T cells were mapped on the F and G proteins of BRSV [86] while M2, F and N proteins seem to be the most important targets for CD8+ T cells [87].

Following BRSV infection there is an increase in CD4+ and CD8+ T cells in the lungs [88]. The CD8+ cytotoxic T lymphocytes have been shown to play a major role in the recovery from BRSV infection [89, 90]. Similar observations were made after BPIV3 challenge of previously vaccinated calves: the production of neutrophil chemotactic factors by alveolar macrophages and the resulting neutrophil influx into the lungs occurred more rapidly than in the control animals resulting in a more rapid clearance of the virus [91].

A major difference in the immune response against BRSV and BPIV3 is vaccine-enhanced immunopathology that has been observed in calves vaccinated with certain inactivated BRSV vaccines prior to natural [92] or experimental [93, 94] BRSV infection. Similar observations were made in HRSV infected infants that had been vaccinated with a formalin-inactivated HRSV vaccine and subsequently experienced a natural HRSV infection [95]. Numerous studies, both with HRSV and BRSV suggest that a disbalanced cellular immune response is involved in the pathogenesis of this phenomenon, yet the immunological mechanisms are still not completely understood [44]. Such an immunopathological phenomenon has never been reported for BPIV3.

#### How to get the right diagnosis?

The importance of early BRD detection is generally acknowledged. Measuring of the body temperature [96, 97], auscultation, ultrasonography [98] and detection of acute phase protein [99] have been described as suitable methods for diagnostics based on clinical signs, yet they do not enable distinction between different respiratory pathogens.

Involvement of BRSV or BPIV3 in an outbreak of BRD can either be determined based on the detection of the virus or by measuring virus-specific antibodies.

Regarding virus detection, it is important to realize that the viruses are only shed during a limited timeframe. BRSV RNA was detected in nasal swab samples starting on day one to day five after experimental infection for up to 4 weeks [100], while previous studies have concluded that viral shedding usually begins later, and lasts for a shorter period [38, 79, 81, 101, 102]. The explanation for this discrepancy is likely related to the difference in detection methods i.e. RNA detection by polymerase chain reaction (PCR) as opposed to virus titration assay in tissue culture in the latter studies. Also, for BPIV3, virus detection in nasal swab samples was positive at an earlier time point and continued for a longer period when tested by PCR as compared to the results of the virus titration in tissue culture (Makoschey et al. unpublished observations).

In the culture method, which could be considered the gold standard, samples are incubated on susceptible cells and virus infection is determined by cytopathic effect or immunostaining using labelled specific antibodies or antiserum. In the case of BPIV3, the culture plates can also be incubated with erythrocytes and subsequently read for haemadsorption.

Due to the nature of the test, the titration method only detects infectious virus particles, while also non-infectious virus particles can lead to a positive PCR result. As both viruses are very labile and easily killed, samples might lose infectivity during transport and storage. Those samples are then found (false) negative in the virus titration assay, but positive in the PCR test. Moreover, also in samples harvested from in vitro cultures BRSV virus titers were significantly lower than RNA copy numbers [103]. As the pattern of values obtained from the two assays over the infection time course correlated closely, the results suggest that, incomplete viral genomes that occur during virus replication might also contribute to the difference between infectious virus titer and RNA copies.

Prior to the widespread application of PCR methods in veterinary diagnostic laboratories, commonly used methods for the detection of BRSV and BPIV3 were fluorescent antibody testing (FAT) on frozen tissue sections [104–106], lung lavage samples [107] or nasal swab samples [108, 109] and Enzyme-Linked Immunosorbent Assay (ELISA) testing of organ homogenates [110]. In general, PCR testing has a better sensitivity than the traditional methods like virus isolation, ELISA and FAT [111, 112]. Currently, different multiplex formats that allow testing of BRSV and BPIV3 together with other agents within the BRD complex have been developed. Some of those are commercially available [113–116]. The testing is commonly applied on nasal swab samples, transtracheal aspiration or bronchoalveolar lavages (BAL). When comparing different sampling methods, results obtained from nasal swabs or BAL were in moderate agreement [117]. BRSV levels in BAL samples from experimentally infected animals were found to be slightly higher than levels in nasal swab samples taken at the same day (Makoschey et al. unpublished observations). Moreover, BAL samples might provide more reliable results for diagnostics of bacterial infections [118].

When testing calves that have been administered an intranasal vaccine, caution must be taken for the interpretation of results as the virus can be detected for more than a week after vaccination [119, 120] and the signal can be derived from vaccine virus or from a mix of wild type and vaccine virus.

As for other viruses, also for BRSV and BPIV3 the traditional methods of antibody testing by neutralization test, complement fixation and in the case of BPIV3, also haemagglutination inhibition (HI) have been described [106, 121–124].

Several ELISA tests for detection and quantification of BRSV and BPIV3 antibodies have been described [125–127]. The ELISA technique is fast, cost-effective, large numbers of samples can be handled, the method can be standardized and as opposed to virus neutralization test, an antibody ELISA does not require handling of live virus. Moreover, the isotype- and subclass of the antibodies can be determined using the ELISA technique [76, 77, 128].

In naïve animals, primary infection with BRSV or BPIV3 can be confirmed using serum samples collected 5–10 days after the appearance of the clinical signs of disease [76, 77]. However, as the viruses are endemic in most herds, the diagnostic value of single serum samples are highest for IgM and IgA that are indicative of a recent (re-)infection [76].

Several commercially available ELISAs used in routine diagnostics and research can be used in serum or milk, and levels of antibodies in serum correlates well with levels of antibodies in milk in individual cows, although the antibody titers are generally lower in milk than in serum [129]. However, the levels of antibodies in bulk tank milk can remain high for several years, and this limits the ability to use the bulk tank milk to determine evolution of disease within a farm [130].

On the other hand, a negative test result in serum or (bulk milk samples) can be used to exclude BRSV or BPIV3 as potential cause in a BRD outbreak on a particular farm.

In herds with recurrent disease, paired samples might be useful to establish a diagnosis. An increase in titer of at least four-fold is considered indicative for an infection. The fact that calves that become infected in the presence of passively derived antibody may not seroconvert [75] should be taken into consideration for the interpretation of results.

In addition to the diagnostic purposes, antibody testing of serum samples taken from calves at the arrival in a fattening unit can provide useful information for the prediction of the risk to develop BRD later in life [131, 132].

Another application might be the monitoring of the immune response after vaccination. In this case, the type of vaccine must be taken into account. In a direct comparison of an inactivated BRSV-BPIV3-*M. haemolytica* vaccine and a modified live BRSV-Bovine Viral Diarrhoea vaccine, the neutralizing antibody profiles were similar, while the antibody levels measured by ELISA were higher for the group vaccinated with the inactivated vaccine [83]. Also, the route of vaccination influences the antibody response. As mentioned earlier, especially live BRSV vaccines applied via the intranasal route have been shown to be efficacious even in the absence of detectable levels of serum antibodies [38, 79–81], Makoschey et al., unpublished observation).

Last but not least, it should be mentioned, that metabolomic profiling might offer new approaches to determine markers for the systemic immune response [133] following virus infection or vaccination.

### Measures against the disease

#### Treatment of sick animals

As for other virus infections, treatment of BRSV and BPIV3 infected animals is mostly limited to supportive measures to keep the affected animals well hydrated and to maintain proper energy and electrolyte balance. If the affected animals do not recover, and the involvement of secondary bacterial infections has been diagnosed, treatment with antimicrobials, for which the bacteria are susceptible, may be required. Furthermore, anti-inflammatory medications can reduce fever, reduce damaging inflammatory response in the lungs and improve the animal’s welfare and thereby feed and water intake. Corticosteroids are not recommended for use in the treatment of BRD due to their immunosuppressive nature. Non-steroidal anti-inflammatory drugs (NSAID) are preferable. Promising results with a combination of antiviral and nonsteroidal anti-inflammatory treatment have recently been obtained in a bovine model of respiratory syncytial virus infection [134].

#### General preventive measures

Vaccination is the most efficacious preventive measure to control BRSV and BPI3V and will be discussed in more detail below.

In addition, general measures should be taken to minimize risk factors for the development of BRD including ensuring optimized environmental conditions [135] and reduction of stress factors [136].

Basic cleaning and hygiene procedures should be applied to prevent or at least reduce the infection pressure. As both viruses have a low tenacity, they are readily inactivated with common disinfectants.

Direct transmission from infected animals, indirect transmission by individuals visiting farms vectoring the viruses [137] or not providing boots for visitors [138] have been identified as risk factors for inter-herd transmission of BRSV. On the other hand, herds can remain seronegative despite proximity to seropositive herds if herd biosecurity is appropriate [139]. Biosecurity measures are also the most important tool within the Norwegian control program for BRSV and Bovine Coronavirus [140].

Good colostrum management is an important preventative measure as low levels of IgG in general and low levels of BRSV specific antibodies were found to be associated with a higher risk of BRD [131].

Novel approaches to BRD disease control and prevention that are currently investigated are innate immunomodulation [141] and the identification of genes and chromosomal regions that underly genetic variation in disease resistance and response to vaccination. Analysis of the genetic variation of animals in a BRSV infection trial suggest that certain motifs in genes related to immunity were associated with high or low antibody and T cell responders [142]. Eventually, this research could lead to selection of animals that are more resistant to disease caused by BRSV and BPIV3 and open new ways to improve vaccine efficacy.

### Vaccination against BRSV and BPIV3

#### Traditional vaccines

Shortly after the discovery of BPIV3, the first inactivated vaccines against this virus were developed [143] followed some years later by modified live virus (MLV) vaccines [144].

Due to the observation of disease enhancement in children vaccinated with a formalin-inactivated HRSV vaccine [145] attempts to develop a BRSV vaccine initially focused on live vaccines [146]. Some years later, promising results were achieved with a BRSV vaccine derived from glutaraldehyde-fixed cells, which did not cause disease enhancement, but even provided better protection than two live-attenuated vaccines tested in the same study [147]. Several inactivated BRSV vaccines have been available and widely used since then, and only incidentally severe courses of BRSV infection have been reported in calves that had previously been vaccinated with formalin-inactivated vaccines [93, 148].

An incident of vaccine associated disease enhancement has also been reported for a beta-propriolactone-inactivated, alum- and saponin-adjuvanted BRSV vaccine [92]. The results from a field trial with a similarly formulated vaccine of which the identity was not disclosed suggested a failure to protect and this vaccine was withdrawn from the market in the late 1990’s [53].

It should be noted that vaccination with a modified live vaccine during the course of a natural infection may also enhance the severity of disease [149].

Interestingly, vaccine associated disease enhancement has only been reported for BRSV and HRSV vaccines, but not for BPIV3 and HPIV3. The immunological mechanisms have not been fully unraveled, but it has been proposed that the inactivation process is able to alter BRSV epitopes and thus the induction of cytotoxic T lymphocyte activity [150] and functional antibodies [151]. This can lead to high levels of non-neutralising antibodies in combination with relatively low levels of neutralising antibodies [148, 152] and increased levels of IgE [153]. Moreover, it has been observed that Interferon gamma production following BRSV infection is reduced in calves previously vaccinated with formalin-inactivated BRSV [154].

Several commercially available live attenuated BRSV and BPIV3 vaccine strains have been obtained using traditional approaches such as passaging in cell culture [146] or selection of temperature-sensitive mutants [78, 155]. In general, the mechanisms of attenuation are unknown, but in a recent study it was shown that transcriptions of cytokines related to fever and inflammation were not upregulated in the nasopharyngeal mucosa after vaccination with a new live attenuated intranasal BRSV-BPIV3 combination vaccine, while these factors were upregulated after infection with BPIV3 field virus [47].

Also, the “Jennerian” approach of using an antigenetically related virus from another species has been tested in the past when calves were vaccinated with a temperature sensitive HRSV strain [147] or a BPIV3 vaccine was evaluated in infants and children [156].

#### Experimental vaccines

Multiple approaches using contemporary vaccine technologies have been investigated with the intention to develop better vaccines for use in cattle, or to use the bovine viruses in their natural host as model for vaccines against their counterpart in humans. The number of approaches is higher for BRSV compared to BPIV3, probably because improved vaccines for use in young children and elderly people are still needed for HRSV while there is less need for new HPIV3 vaccines.

Subunit vaccines based on the major glycoproteins of BRSV have been tested with good results, both for parenteral [80, 157] and intranasal application [158]. After intranasal application of BPIV3 antigen formulated in nanoparticles, a mucosal IgA response was measured [159], yet protection against infection was not tested.

The development of a reverse genetic system for BRSV [160] enabled the engineering of recombinant viruses. Several viruses lacking one or more proteins induced at least partial protection in calf models [7, 11, 17, 80]. A further development of a BRSV virus lacking the G and the F protein was a chimeric virus in which these proteins were replaced by BPIV3 HA and F protein [8]. Such a virus might potentially be a bivalent vaccine against both, BRSV and BPIV-3, but to our best knowledge, this has not yet been demonstrated in calves.

Promising results with human recombinant RSV-vaccine candidates in which the F glycoprotein is stabilized in its prefusion state could be reproduced with recombinant BRSV in the calf model initially in animals without maternal antibodies [161] and more recently also in animals with maternal antibodies [162].

Last but not least, chimeric vaccinia viruses [163, 164] or bovine herpesviruses [165] expressing BRSV proteins have been developed as vaccine candidates. One major advantage of these viruses for vaccine development would be that they grow much better in cell culture than the BRSV viruses.

Another advantage is that some of these vaccines offer DIVA properties, which allow to differentiate between infected and vaccinated animals by serological testing. Such vaccines would be helpful for monitoring efficacy of biosecurity measures or for countries with BRSV control programs such as Norway.

Although the results obtained with several of the above-mentioned vaccine candidates were promising, none of them clearly outperformed the currently available commercial vaccines with regards to all the requirements for yields, process robustness, safety and efficacy.

Completely novel approaches to vaccine development might become available in the future thanks to the progress in the understanding of host pathways involved in the innate anti-viral response, together with the capability to generate substances that can interfere with these processes [166].

#### Efficacy testing of commercial vaccines

Prior to commercialization, the efficacy of any new vaccine must be demonstrated, under both experimental and field conditions as prescribed in relevant regulations. Given the multifactorial nature of the disease, it is rather demanding to reproduce clinical signs under experimental conditions [167]. A comprehensive overview of the literature concerning challenge models for BRSV, BPIV3 and other common BRD pathogens was prepared by Grissett and colleagues [168].

In the first infection studies with cell-culture-passaged BRSV only mild disease or no disease at all was observed in the unvaccinated control animals [169], even in colostrum deprived or gnotobiotic calves. The suggestion that the viruses attenuate rapidly upon culture in vitro has been supported by several studies in which clinical signs of respiratory disease were reproduced by inoculation of low-passage BRS virus [20, 170].

Early studies investigated different administration protocols for the BRSV and BPIV3 challenge strains including multiple application [171] and invasive (intratracheal) routes [172, 173], which are not representative of natural transmission. Aerosolization, a delivery method that mimics the natural route of transmission, was found to produce more consistent results [148, 170, 174]. The same method has also been successfully applied in BPIV3 infection studies [175].

Many efficacy studies with commercially available BRSV and BPIV3 vaccines both under experimental and field conditions have been published and comprehensive reviews are available [167, 176]. Most of these studies estimated clinical efficacy from results of experimental challenge studies. Interpretation of the results requires caution as some of the models are not representative for natural exposure.

An important requirement for live BRD vaccines is an early onset of immunity. Studies with a live marker vaccine against Bovine Herpesvirus have shown that the animals were protected as early as 3 days after intranasal vaccination [177]. Studies to determine the onset of immunity of the currently available BRSV and BPIV3 vaccines were hampered by the fact that the commonly applied methods for virus detection in nasal discharge do not discriminate between vaccine and field strains. By consequence, vaccine virus interferes with the detection of wild-type virus if the experimental infection is done too shortly after vaccination. For intranasal BRSV-BPIV3 combination vaccines commercialized in Europe, an onset of immunity was seen 5 days after vaccination with regards to BRSV [175, 178] and 7 [175] or 10 days [155] with regards to BPIV3.

The onset of immunity has also been studied for an inactivated BRSV-BPIV3-*M. haemolytica* vaccine. A single dose was shown to prime the cellular immune response in calves around 2 weeks of age with maternal antibody [85] and provided partial protection against experimental BRSV infection [179], yet complete protection can only be expected after completion of the two-dose vaccination course. Moreover, it should be noted, that not all inactivated BRSV-BPIV3 vaccines have demonstrated protection in face of maternally derived antibodies.

In field studies, efficacy is typically evaluated by general parameters for disease such as mortality, morbidity, treatments and growth rate while no or only limited information is available about the involvement of specific pathogens in the disease outbreak. Several studies in which commercially available MLVs with and without BRSV were compared, indicated a reduction of respiratory disease [180, 181], or improved (milk) production and reproductive parameters [182] in the groups vaccinated with BRSV. In a recent field trial performed with a new BRSV-BPIV3 live vaccine for intranasal use, the prevalence of eight different BRD pathogens was monitored by PCR testing of nasal swab samples. BRSV infection occurred in several farms, and the nasal shedding of BRSV was significantly lower in the vaccinated animals [183].

#### How to make best use of BRSV and BPIV3 vaccines

In our current production systems young calves are assembled under stressful conditions in high numbers, which at the same time increases the infectious pressure and weakens the immune system of the calves. Early in live, calves depend on the colostral immunity for protection against infectious agents. Unfortunately, the amount of specific maternal antibodies is very variable and the duration of protection by colostral antibodies is difficult to predict. By consequence, vaccines must be applied early in live and have an early onset of immunity to protect those calves that have received low levels of colostral antibodies. On the other hand, the vaccines should also be efficacious in the face of maternal antibodies (IFOMA) to provide immunity to those calves that have received high levels of colostral antibodies.

The first commercially available BRSV and BPIV3 vaccines (live and inactivated) were licensed for parenteral use. The difficulties and opportunities for vaccinating calves IFOMA have recently been reviewed by Windeyer and Gamsjäger [184]. They concluded that parental vaccination IFOMA is unlikely to result in seroconversion, and other immune responses are inconsistent, but the presence of antibodies may be prolonged and immunological memory might be induced. Moreover, reduction of clinical signs was reported by Chamorro and colleagues [185].

The potential advantages of intranasal vaccination with a live vaccine IFOMA by stimulation of a local immune response and priming the systemic immune response prompted Ellis and colleagues to determine the efficacy of a live vaccine for parental delivery after intranasal administration [81]. That study suggested that similar levels of protection were provided by intranasal and parenteral administration. However, it should be noted, that the calves in that study had low levels of colostral antibodies.

Nowadays, several MLV BRSV and BPIV3 vaccines for intranasal application are commercially available. Typically, spraying devices generating a kind of aerosol are required for administration of these vaccines, however, in a recent study with a new BRSV-BPIV3 live vaccine, animals vaccinated without spraying device (directly from the tip of the syringe) were protected against experimental BRSV and BPIV3 infection [186].

An alternative approach to protect the calf early in life is the vaccination of the pregnant dam to achieve higher and more homogenous levels of antibodies in the colostrum [187, 188] and also specific memory cells in the calves [189]. Calves fed colostrum from vaccinated dams were partly protected against BRSV infection [73]. Therefore, cow vaccination in combination with good colostrum management might be considered to complement an active immunisation program against BRD.

Given the involvement of multiple different pathogens in BRD, an important selection criterion for a vaccine is the range of antigens against which protection is provided. Multivalent vaccines have been available since more than three decades [190] and most commercially available BRSV and BPIV3 vaccines contain both viruses together with one or more other viruses and/or bacteria. In comparative field trials an inactivated BRSV-BPIV3-*M. haemolytica* vaccine provided better protection against BRD than MLV BRSV-BPIV combination vaccines [191, 192]. These observations illustrate the fact that BRD outbreaks in the field are often a combination of viral and bacterial pathogens.

Several studies have been performed to investigate the possibility to combine BRSV-BPIV3 vaccines with other vaccines, for example the combination of a live BRSV-BPIV3 vaccine with an *M. haemolytica* vaccine [193] or the combination of an inactivate BRSV-BPIV3-*M. haemolytica* vaccine with a live Bovine Herpesvirus vaccine [194] or an inactivated vaccine against neonatal diarrhea [195].

A general concern especially with BRSV vaccines is the rather short duration of immunity as compared to other viruses [79, 101, 175, 178, 196]. The observation that re-infections are common [65] suggest that also the immunity following field infection is of relatively short duration. Therefore, re-vaccination of animals is advised to achieve lasting herd immunity [197].

The timing of vaccination/re-vaccination can also have a direct impact on the clinical benefit [198]. In a comparative study, vaccination of calves with an inactivated BRSV-BPIV3-*M. haemolytica* vaccine prior to transport to the fattening units resulted in better protection against BRD than vaccination in the fattening unit [199]. In the current complex economic structure of the cattle industry, cow-calf producers often do not have economic incentive to vaccinate the calves [200]. This might change in the future if technologies to unambiguously identify properly vaccinated animals become available.

Different re- vaccination schedules have been investigated including e.g. the antibody response after a single booster vaccination with an inactivated BRSV-BPIV3-*M. haemolytica* vaccine given up to 12 months after completion of the primary vaccination course was found to be similar than the levels after the primary vaccination course [201].

Good results in terms of protection against experimental BRSV infection were obtained with a combined vaccination schedule of a primary vaccination course with a live vaccine applied intranasally followed by parental application of an inactivated vaccine [202]. On the other hand, an intranasal booster dose of a BPIV3 following a priming by the subcutaneous route produced slightly better protection than the subcutaneous dose alone [203].

#### Control of BRSV and BPIV3

In Norway, a program to control BRSV and Bovine Coronavirus was initiated [140] with monitoring and biosecurity measures as the main tools. A similar approach has given good results in the control of Bovine Viral Diarrhoea Virus (BVDV) and Bovine Herpesvirus Type 1 (BHV-1). The success of the latter control programs in Nordic countries had prompted other European countries to also embark in control initiatives for these viruses [204], but in most countries, vaccination is also used as a tool. Initially, the prevalence of BVDV and BHV1 remained more or less constant although vaccines were available. Significant progress in the control of these viruses was only achieved once vaccination was applied widely and in a systematic matter.

Currently, the vaccine coverage for BRSV and BPIV3 is rather low: A survey of cattle farmers in Ireland and the UK revealed that two-thirds of the farmers do not vaccinate at all and only 20% or 7% vaccinate all calves retained/brought onto the farm under 3 and 9 months of age, respectively [205], similar results have been obtained for other countries (Vertenten unpublished data). Such a low vaccination rate is unlikely to lead to a reduction of the prevalence of the two viruses.

On herd level, the best benefit of the vaccines can be achieved with a tailormade herd immunisation program which addresses all relevant herd-specific aspects such as the age distribution and origin of the animals, the epidemiological situation and the level of maternal immunity. For example, early calfhood vaccination is particularly important in herds with poor passive immunity, but it should be taken into consideration, that the immunological changes during the first few weeks of a calf’s life [206] and nutritional deficiencies [207] might negatively affect the level of protection that can be achieved by vaccination.

## Conclusion

BRSV and BPIV3 are important pathogens in cattle and related to outbreaks of respiratory disease. The two viruses share a lot of morphological and biological characteristics between each other as well as with their counterparts in humans, HRSV and HPIV3. Intense studies on BRSV and BPIV3 have not only lead to the development of vaccines for use in cattle, but also improved our understanding of the disease in cattle and humans. Based on this knowledge, we can conclude that the viruses BRSV and BPIV3 are only two out of multiple factors that lead to BRD. Especially in some of our production systems where we assemble high numbers of calves from various sources under stressful conditions, the BRD problem can only be solved by a holistic approach in which systematic vaccinations programs, preferably also at the herd of origin are supported by state-of-the-art herd management and biosecurity measures.

## Data Availability

Not applicable.

## Abbreviations

BAL: Bronchoalveolar Lavage
BHV-1: Bovine Herpes Virus 1
BPIV3: Bovine Parainfluenza 3 Virus
BRD: Bovine Respiratory Disease
BRSV: Bovine Respiratory Syncytial Virus
BVDV: Bovine Viral Diarrhoea Virus
CD: Cluster of Differentiation
DIVA: Differentiating Infected from Vaccinated Animals
ELISA: Enzyme Linked Immunosorbent Assay
F: Fusion protein
FAT: Fluorescent Antibody Technique
G: G protein
HI: Haemagglutination Inhibition
HN: haemagglutinin-neuraminidase
HPIV3: Human Parainfluenza 3 Virus
HRSV: Human Respiratory Syncytical Virus
IFN: Interferon
IFOMA: In Face Of Maternal Antibodies
IgA: Immunoglobulin A
IgE: Immunoglobulin E
IgG: Immunoglobulin G
IgM: Immunoglobulin M
IL: Interleukin
L: Polymerase protein
M: Matrix protein
MLV: Modified Live Virus
N: Nucleoprotein
NMBU: Norwegian University of Life Sciences
NS: Non-Structural
NSAID: Non-Steroidal Anti-Inflammatory Drug
P: Phosphoprotein
PAM: Pulmonary Alveolar Macrophages
PCR: Polymerase Chain Reaction
RNA: Ribonucleic Acid
mRNA: messenger RiboNucleic Acid
SH: Small Hydrophobic Protein
TNF: Tumor Necrosis Factor
